# Associations between human milk EV-miRNAs and oligosaccharide concentrations in human milk

**DOI:** 10.3389/fimmu.2024.1463463

**Published:** 2024-11-20

**Authors:** Elizabeth A. Holzhausen, William B. Patterson, Benjamin H. Wong, Sewan Kim, Allison Kupsco, Caitlin G. Howe, Lars Bode, Michael I. Goran, Tanya L. Alderete

**Affiliations:** ^1^ Department of Integrative Physiology, University of Colorado Boulder, Boulder, CO, United States; ^2^ Department of Environmental Health and Engineering, Johns Hopkins Bloomberg School of Public Health, Baltimore, MD, United States; ^3^ University of Colorado Anschutz Medical Campus, Aurora, CO, United States; ^4^ Department of Environmental Health Sciences, Columbia University Mailman School of Public Health, New York, NY, United States; ^5^ Department of Epidemiology, Geisel School of Medicine at Dartmouth, Lebanon, NH, United States; ^6^ Department of Pediatrics, Larson-Rosenquist Foundation Mother-Milk-Infant Center of Research Excellence (MOMI CORE), Human Milk Institute (HMI), University of California, San Diego, San Diego, CA, United States; ^7^ Department of Pediatrics, Children’s Hospital Los Angeles, Los Angeles, CA, United States

**Keywords:** microRNA, human milk (HM), human milk oligosaccharides, extracellular vesicles, EV-microRNAs

## Abstract

**Introduction:**

Human milk contains human milk oligosaccharides (HMOs) and microRNAs (miRNAs), which are key bioactive components. HMOs are indigestible carbohydrates that impact infant growth and development. miRNAs are small, non-coding RNAs that regulate post-transcriptional gene expression. miRNAs are abundant in human milk and can be contained in extracellular vesicles (EVs). There is evidence that miRNAs are synthesized in the mammary epithelium and may influence mammary gland development and milk synthesis. However, the relationships between miRNAs and HMOs have yet to be fully characterized.

**Methods:**

This study examined the associations between 210 human milk EV-miRNAs and 19 HMOs in a cohort of 98 Latina mothers. HMO measures included summary measures and concentrations of 19 HMOs. Relationships between EV-miRNAs and HMOs were examined using principal components analysis and associations between individual EV-miRNAs and HMOs were assessed.

**Results:**

Overall patterns of EV-miRNA levels, summarized using principal components, were associated with HMO summary measures and concentrations. Levels of individual EV-miRNAs were associated with HMO summary measures and individual concentrations of 2’FL, 3FL, 3’SL, 6’SL, FLNH, LNFP I, and LNH.

**Discussion:**

Results from this study suggest that human milk EV-miRNAs are associated with the concentration of HMOs, which may have important effects on infant growth and development.

## Graphical Abstract

Images created with Dalle AI and BioRender.com.

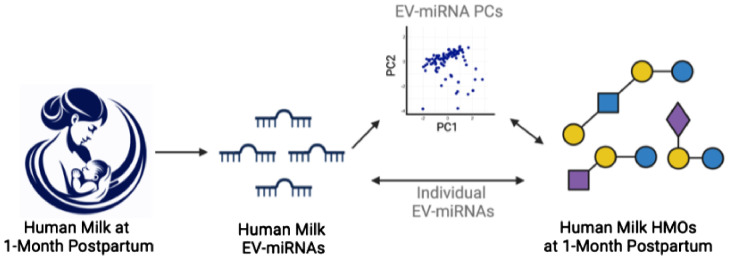

## Introduction

Human milk is the optimal source of nutrition for term infants and is associated with a wide variety of positive health outcomes (1–8). Human milk oligosaccharides (HMOs) are indigestible carbohydrates (9, 10) which are found in colostrum, transitional, and mature milk. HMOs are the third-most abundant solid component of human milk and are composed of five fundamental monosaccharides: glucose, galactose, N-acetylglucosamine, fucose, and sialic acid. The structure of nearly all HMOs features lactose at their reducing end (11). HMOs resist digestion to have prebiotic effects in the infant gut (12–14), serve as antiadhesive antimicrobials (15, 16), mediate epithelial cell responses (14, 17), are immune modulators (18), and support brain development (19). Additionally, HMO diversity, a summary measure of overall richness and evenness of HMO levels, has previously been associated with child growth measures including with child height and weight (20). Nevertheless, numerous aspects of HMO biosynthesis have yet to be fully characterized.

HMO diversity and concentrations of specific HMOs are primarily determined by genetics, and genetic variation can explain up to 55% of variability in HMO concentrations (21). For example, individuals with an active *Se* locus are defined as Secretors. These individuals produce milk abundant in 2’-fucosyllactose (2’FL), Lacto-N-Fucopentaose I (LNFP I), and other alpha1-2-fucosylated HMOs. In contrast, individuals without an active *Se* locus lack the FUT2 enzyme and their milk contains few to no alpha1-2-fucosylated HMOs (9). Similarly, the Lewis gene regulates expression of Le^a^ and Le^b^ antigens. Human milk from individuals without enzymes from the Lewis gene produce milk with low LNFP II (22). In addition to genetics, HMO concentrations change throughout lactation (23–25). Though the highest concentrations of most HMOs are present at the colostrum stage, the concentration of some HMOs increase with infant age (23, 26). Further, HMO concentrations have been associated with season and geographic location (which may be closely linked with genetics) (10, 27), maternal body mass index and obesity (10, 24, 28, 29), maternal age (10, 24), parity (27), ethnicity (27), and exclusive breastfeeding (27). Despite the identification of these factors that influence HMO concentrations, their biosynthesis and determinants of their concentration have not yet been fully elucidated.

In addition to HMOs, human milk contains several other bioactive components, which include hormones, cytokines, leukocytes, immunoglobulins, and microRNAs (miRNAs) (30). miRNAs are small, non-coding RNAs that regulate gene expression post-transcriptionally. Human milk is one of the most abundant sources of miRNAs and there is evidence that these miRNAs are synthesized in the mammary epithelium (31) and may influence mammary gland development (32). miRNAs can be contained inside of extracellular vesicles (i.e., EV-miRNAs), which confer resistance to harsh environments. Importantly, EV-miRNAs can survive digestion *in vitro* (33–36) and may be taken up by epithelial cells in the infant gut (35, 36). Therefore, EV-miRNAs in human milk may impact postnatal gut maturation, nutrient uptake, and infant health via local regulation of post-transcriptional gene expression. Similar to HMOs, human milk miRNAs have been shown to vary by gestational age and pre-pregnancy BMI (37, 38) and may also be influenced by breastfeeding (39). Importantly, many of the miRNAs that are abundant in human milk are involved in milk synthesis (e.g., triacylglycerol in milk fat) (39). However, whether human milk miRNAs may also influence HMO composition in human milk is currently unknown.

Although HMOs are essential for infant nutrition and health, the factors that determine HMO concentrations remain incompletely understood. We hypothesize that human milk EV-miRNAs influence HMO concentrations. This hypothesis is supported by the observation that miRNAs play a critical role in regulating gene expression at the post-transcriptional level and many shared factors that affect miRNA abundance are also linked with HMO concentrations, such as maternal BMI and breastfeeding (10, 24, 27–29, 37–39). Given this, the primary aim of this study was to determine whether human milk EV-miRNAs at 1-month postpartum are associated with summary measures of HMOs (i.e., diversity, sum of all HMOs, and sum of HMO-bound sialic acid and HMO-bound fucose) and concentrations of 19 HMOs that our analytical platform can quantify with confidence.

## Methods

### Study participants

The Mother’s Milk Study is a longitudinal cohort of Latino mother-infant dyads from Southern California, which is investigating the associations between HMOs, the infant gut microbiota, and early life growth and development. Detailed methods have been previously described (40, 41). Briefly, participants were recruited from Los Angeles County maternity clinics affiliated with the University of Southern California. Individuals were eligible to participate if they were 18 years of age or older at time of delivery; had a healthy, singleton birth; were enrolled by approximately one month postpartum; and were able to read at the 5^th^ grade level in either Spanish or English. Individuals were excluded if they had any medical diagnoses or were taking medications known to affect physical or mental health, nutritional status, or metabolism; were current tobacco users (>1 cigarette in the past week); reported recreational drug use; had pre-term or low birth weight infants; or had infants with any clinically diagnosed fetal abnormalities. The Institutional Review Boards of the University of Southern California, Children’s Hospital of Los Angeles, and the University of Colorado Boulder approved study procedures. Written informed consent was obtained from participants prior to study enrollment.

### Study design

Participants had visits at 1-, 6-, 12-, 18-, and 24-months postpartum. The Mother’s Milk Study had 219 mother-infant dyads, 209 of whom contributed a human milk sample at the 1-month visit. Of these, funding was available to assess EV-miRNA levels in 110 human milk samples. Among these 110, 11 were removed since they were classified as HMO “non-secretors” (produced under 500 nmol/mL of the HMO 2’FL). We elected to exclude non-secretors because of their low abundance in our sample. An additional participant was excluded as they produced an intermediate amount of 2’FL (559 nmol/mL, considered a “non-secretor” by some definitions), and had HMO diversity 3 standard deviations (SD) from the mean. This resulted in a final sample size of 98. Participants who were excluded from EV-miRNA analysis were similar to those who were included (40).

### Clinical assessments

The clinical measures used for this study were derived from the 1-month postpartum visit. Maternal weight (kg) was measured using an electronic scale and standing height was measured using a stadiometer (m) to calculate maternal body mass index (BMI, kg/m^2^). Additionally, maternal age at delivery, infant sex, delivery mode, days postpartum, and number of breast feedings per day were collected at the 1-month postpartum visit. Questionnaires were used to determine breast feedings per day, where participants selected 0-1, 1, 2, 3, 4, 5, 6, 7, or ≥ 8 feedings/day and this was treated as a continuous variable. Non-consecutive 24-hour dietary recalls were performed to represent average maternal dietary intake, and from this, the healthy eating index (HEI) was calculated (42). HEI is a composite dietary measure which assesses how well dietary intake aligns with the Dietary Guidelines for Americans (43). Socioeconomic status was measured using a modified version of the Hollingshead Index as previously reported (44).

### HMO analysis

Mothers fasted for at least 1 hour before human milk samples were collected, and sample collection occurred at least 1.5 hours after the most recent feeding. Participants were instructed to provide a single full expression from the right breast using an electric breast pump, ensuring the collection of fore-, mid-, and hind-milk as previously described (45). Milk was frozen and stored at -80°C until analysis. The Bode Lab at the University of California San Diego conducted HMO analysis. For each participant, one 500μL aliquot of human milk was shipped on dry ice. HMOs are extremely stable, and do not degrade during repeated freeze/thaw cycles or pasteurization (46). The HMO analysis has been previously described in detail (23). Briefly, HPLC after fluorescent derivatization (Vanquish Quaternary HPLC with fluorescent detection, Thermo Fisher Scientific) was used for HMO analysis. Lipids, proteins, salts, etc., were removed using solid phase extraction over C18 and Carbograph. Next, the reducing end of oligosaccharides were labeled with 2-aminobenzamide and removed excess label by solid phase extraction over silica. To account for analyte loss during the extraction procedure, raffinose was added at the beginning of sample processing. Overall, 19 HMOs were identified and quantified: 2′-fucosyllactose (2’FL), 3-fucosyllactose (3FL), 3′-sialyllactose (3’SL), 6′-sialyllactose (6’SL), difucosyllactose (DFLac), difucosyllacto-*N*-hexaose (DFLNH), difucosyllacto-*N*-tetrose (DFLNT), disialyllacto-*N*-hexaose (DSLNH), disialyllacto-*N*-tetraose (DSLNT), fucodisialyllacto-*N*-hexaose (FDSLNH), fucosyllacto-*N*-hexaose (FLNH), lacto-*N*-fucopentaose (LNFP) I, LNFP II, LNFP III, lacto-*N*-hexaose (LNH), lacto-*N*-neotetraose (LNnT), lacto-*N*-tetrose (LNT), sialyl-lacto-*N*-tetraose b (LSTb), and sialyl-lacto-*N*-tetraose c (LSTc). These are 19 of the most abundant HMOs, and they represent more than 95% of the total HMO concentration and all structural features of HMOs, including chain elongation, branching, and all known types of fucosylation and sialylation. HMO concentrations are reported in nmol/mL. HMO diversity was estimated using Simpson’s diversity. The summary measure, sum of all HMOs in a sample, is the sum of all HMOs quantified in each sample. HMO-bound fucose and HMO-bound sialic acid are the sum of all sialic acid and all fucose molecules bound to HMOs in a sample, respectively (e.g., each molecule of 2’FL contains 1 molecule of fucose, and each molecule of DFLNT contains 2 molecules of fucose).

### EV-miRNA sequencing, processing, and expression

EVs were isolated from stored human milk samples as previously described (47). Milk samples were thawed on ice prior to analyses. Next, samples were centrifuged to remove the lipid layer, then again to remove cellular debris and apoptotic bodies. EVs were extracted using the ExoEasy Maxi KIT (Qiagen, Germantown, MD) and total RNA was isolated with the miRNeasy Serum/Plasma Maxi KIT (Qiagen, Germantown, MD). Samples were cleaned using the RNA Clean & Concentrator-5 Kit (Zymo Research, Irvine, CA) and sample purity and quantity were measured on an Implen NanoPhotometer spectrophotometer (München, Germany). As previously described (40), concentration, sizes, and distribution were assessed in four randomly selected samples using nanoparticle tracking analysis on the ViewSizer 3000 (Horiba Scientific, Piscataway, NJ). The Exo-Check Exosome Antibody Assay (System Biosciences, Palo Alto, CA) was used to confirm the presence and purity of isolated EVs on four sets of three pooled EV samples and three sets of three matched EV-depleted samples. All relevant EV characterization data have been submitted to the EV-TRACK knowledgebase (EV-TRACK ID: EV220416) (48).

Sequencing and library preparation was performed at the University of California San Diego. The NEBNext Small RNA Library Prep Set for Illumina (NEB, Ipswich, MA) was used to construct sequencing libraries with optimization to account for low input and cell-free RNA. Reactions were conducted at one-fifth the recommended volume, adapters were diluted 1:6, and library amplification PCR used 17 cycles. Libraries were cleaned with the DNA Clean and Concentrator Kit (Zymo Research, Irvine, CA) and the concentrations were quantified using the Quant-iT PicoGreen dsDNA Assay (Invitrogen, Waltham, MA). Samples were pooled with equal volumes. The pool’s size distribution was determined with a DNA HS Chip on a BioAnalyzer (Agilent Technologies, Santa Clara, CA) before size selection (115-150 base pairs [bp]) on a Pippin Prep instrument (Sage, Beverly, MA) to remove adapter dimers and fragments larger than the target miRNA population. Libraries were sequenced to ~1 million total reads per pool using a MiSeq instrument with a Nano flow cell (Illumina Inc, San Diego, CA). This sequencing data was used to balance the samples into new pools for deeper sequencing on a HiSeq4000 instrument using single-end 75 bp runs.

Sequencing data were mapped using the ExceRpt small RNA sequencing data analysis pipeline on the Genboree Workbench (49). Samples were mapped using default parameters, except for filtering to a minimum read length of 15 nucleotides with zero mismatches. Quality control was performed according to External RNA Controls Consortium guidelines (49). One sample had <100,000 transcriptome reads and was removed from subsequent analysis. Raw EV-miRNA read counts were normalized using the trimmed mean of M (TMM) method from the EdgeR package (50).

### Statistical analysis

Descriptive statistics were calculated using the mean and standard deviation (SD) for continuous variables and frequencies for categorical variables. We examined the associations between human milk EV-miRNAs and HMOs using two approaches. First, due to the high-dimensional EV-miRNA data, we summarized EV-miRNA levels using principal components (PC) analysis. PCs were then used to assess whether EV-miRNA profiles were associated with HMOs (i.e., diversity, HMO-bound sialic acid, HMO-bound fucose, and individual HMO concentrations). To accomplish this, multivariable linear regression was used to estimate the independent associations between our primary predictors (PC1 and PC2) with HMO measures. Prior to principal component transformation, values were centered and scaled to have unit variance. As a second approach, multivariable linear regression models were used to estimate the associations between individual EV-miRNAs with human milk HMOs. Based on our previous work, all models adjusted for technical covariates (i.e., proportion of rRNA, volume of skim milk) as well as days postpartum, time of day of milk collection, and number of breast feedings per day (40). As a sensitivity analysis, we additionally adjusted for maternal variables age, BMI, and HEI. Unadjusted p-values are reported; however, all analyses were also adjusted for multiple testing using the Benjamini-Hochberg procedure with a threshold of P_BH_ < 0.10 (51).

Pathway analysis was used to characterize putative mRNA targets of EV-miRNAs that were associated with HMO expression using DIANA MirPath version 4 (52). For pathway analysis, we converted all precursor EV-miRNAs into their mature counterparts, which applied only to hsa-mir-378c. Tarbase v8.0 (53), a catalogue of experimentally validated miRNA-gene interactions, and microT-CDS (54), which predicts *in silico* miRNA-gene interactions, were used to identify Kyoto Encyclopedia of Genes and Genomes (KEGG) pathways with significant enrichment.

## Results

### Population characteristics

**Figure 1:**
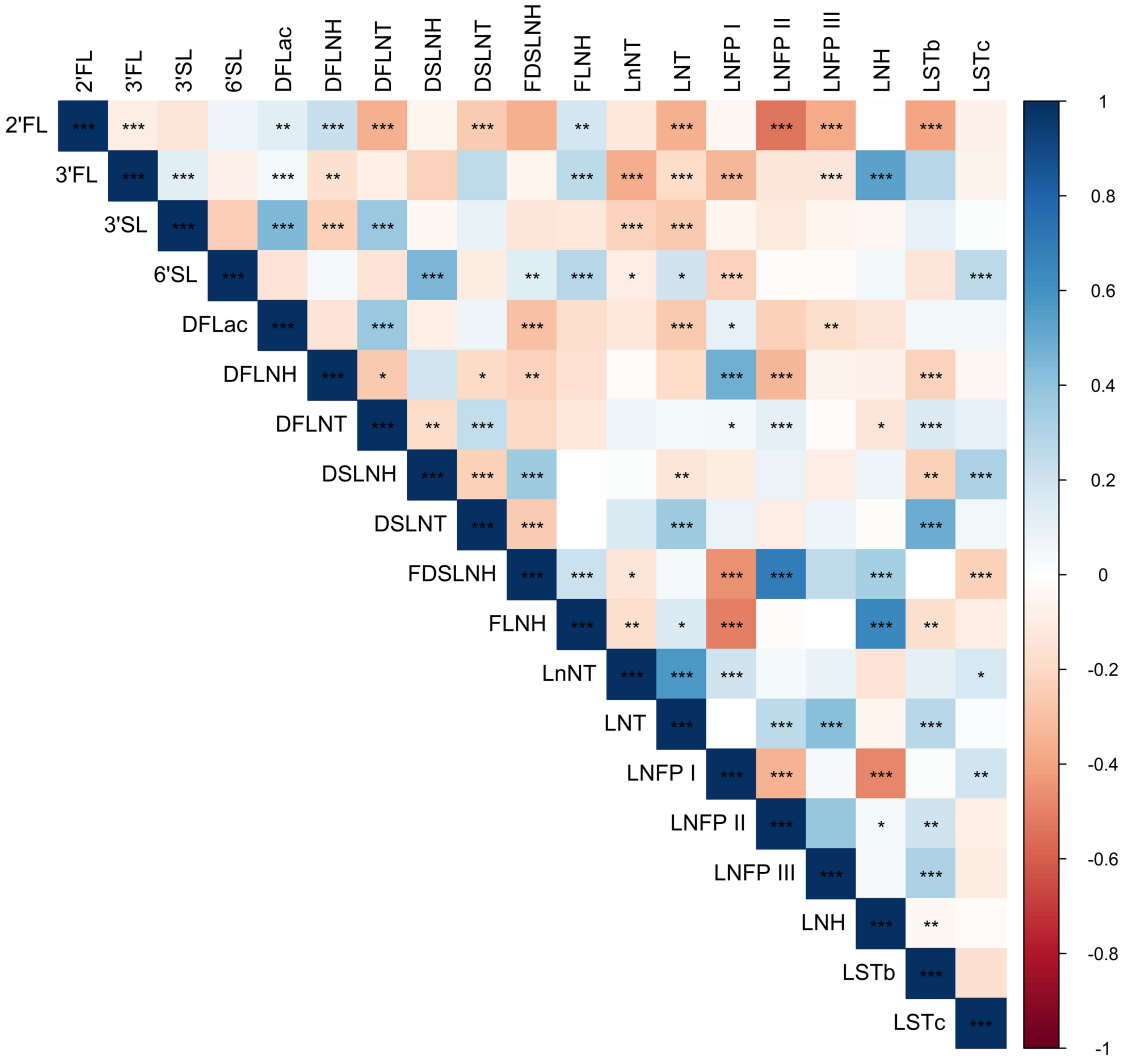
Heatmap showing Spearman correlations among HMO concentrations. Blue shading indicates positive Spearman correlation and red shading indicates negative Spearman correlation; color saturation indicates the strength of the correlation. Stars indicate statistical significance, with *** showing correlations with P < 0.001, ** showing correlations with P < 0.01, and * showing correlations with P < 0.05.

**Table 1** shows the mean physical and social characteristics of the 98 mothers included in the analysis. The Hollingshead Index was used to measure the participant’s socioeconomic status, where 54% had low or very low SES (Hollingshead score < 26.5). On average, mothers were 27.9 years of age at the time of birth (range: 18-42 years) and overweight at 1-month postpartum (BMI: 29.9 ± 4.8 kg/m^2^). Additionally, at 1-month postpartum, 16.3% of mothers had a healthy weight, 38.8% had overweight, and 44.9% had obesity. Mothers self-reported that they were breastfeeding an average of 6.3 times per day (range: 0-8) and were in the mature milk stage where, on average, human milk samples were collected 32.5 days after delivery. The top five most abundant EV-miRNAs were miR-148a-3p, miR-146b-5p, miR-200a-3p, let-7g-5p, and let-7b-5p, which is largely consistent with previous studies (55). These EV-miRNAs were detected in every sample and comprised 15.5% of the total reads. As expected, the concentrations of several human milk HMOs were both positively and negatively correlated with one another at 1-month postpartum (**Figure 1**). **Table 2** shows the characteristics of HMO summary measures and individual concentrations at 1-month postpartum.

**Table 1:** Characteristics of 98 mothers from the Southern California Mother’s Milk study at 1-month postpartum.

Maternal Characteristics	Mean ± SD or N, %
Maternal age at birth (years)	27.9 ± 5.7
Socioeconomic status^a^	26.0 ± 12
Infant sex (female, male, % female)	55, 43, 56%
Delivery (vaginal, caesarean, % vaginal)	75, 23, 76.5%
Days post-partum	32.5 ± 3.1
Number of breastfeedings per day	6.3 ± 2.4
Maternal BMI (kg/m^2^)	29.9 ± 4.8
Health Eating Index	67.9 ± 11.4

Baseline (1-month) characteristics of 98 Latina mothers from the Southern California Mother’s Milk Study. Data are reported as mean and standard deviation (SD) unless otherwise noted.

a SES was estimated using a modified version of the Hollingshead index. Range (3,63).

**Table 2:** Characteristics of HMOs at 1-month postpartum.

HMO Summary Measures (nmol/mL)	Mean ± SD
Diversity*	5.1 ± 1.9
Sum of HMOs	16883.9 ± 1813.0
HMO-bound sialic acid	3431.5 ± 888.4
HMO-bound fucose	14583.0 ± 2036.6
HMO Concentrations (nmol/mL)	Mean ± SD
2’FL	6771.4 ± 3175.6
3FL	748.2 ± 1151.5
3’SL	592.5 ± 658.9
6’SL	956.5 ± 315.9
DFLac	475.6 ± 292.5
DFLNH	159.0 ± 122.2
DFLNT	1491.4 ± 815.2
DSLNH	159.0 ± 122.2
DSLNT	389.1 ± 179.7
FDSLNH	114.2 ± 98.1
FLNH	137.6 ± 112.0
LnNT	625.0 ± 322.7
LNT	1130.1 ± 667.2
LNFP I	1656.7 ± 1056.4
LNFP II	842.4 ± 387.7
LNFP III	60.4 ± 29.1
LNH	92.4 ± 61.4
LSTb	88.1 ± 54.0
LSTc	318.4 ± 152.3

Baseline (1-month) summary of HMOs among 98 Latina mothers from the Southern California Mother’s Milk Study. Data are reported as mean and standard deviation (SD). *Diversity was estimated using Simpson’s Diversity measure.

### EV-miRNA principal components are associated with HMO measures

PC analysis was used to assess whether EV-miRNA profiles were associated with HMO summary measures and concentrations (**Supplementary Figure 1**). Here, we focused on PC1 and PC2, which explained a total of 34.7% of the variation in EV-miRNA measures (**Supplementary Figure 2**). We did not include subsequent PCs because they explained little additional variance (e.g., PC3 explained 5.9%). Using multivariable linear models, we constructed models that included both PC1 and PC2 while adjusting for our *a priori* covariates. PC2 was inversely associated with HMO diversity (P = 0.046), positively associated with the sum of all HMOs (P = 0.0001), and positively associated with HMO-bound fucose (P = 0.02). The EV-miRNAs that were most positively associated with PC2 included miR-183-5p, miR-151a-3p, miR-511-5p, miR-99b-5p, and miR-3615. Conversely, the EV-miRNAs that were most inversely associated with PC2 included miR-502-3p, miR-629-5p, miR-146a-5p, miR-500a-3p, and miR-21-5p (**Supplementary Table 1**). Additionally, PC2 was positively associated with the concentration of three HMOS, 2’FL, 6’SL, and FLNH (**Table 3**, all P_BH_ < 0.10). We did not observe any statistically significant associations between PC1 with HMO measures. PC1 was most positively associated with levels of miR-183-5p, miR-151a-3p, miR-511-5p, miR-99b-5p, and miR-3615 and was most negatively associated with levels of miR-21-5p, miR-500a-3p, miR-146a-5p, miR-629-5p, and miR-502-3p.

**Table 3:** PC2 was associated with several HMO measures.

HMO Summary Measures (nmol/mL)	PC1		PC2	
	β (95% CI)	P	β (95% CI)	P
Diversity^+^	-0.02 (-0.09, 0.05)	0.55	-0.08 (-0.16, -0.001)	**4.6x10^-2^**
Sum of HMOs	11.79 (-52.17, 75.75)	0.72	151.40 (77.57, 225.23)	**1.0x10^-4^**
HMO-bound fucose	10.76 (-63.81, 85.32)	0.78	103.99 (17.91, 190.07)	**0.02**
HMO-bound sialic acid	20.21 (-12.23, 52.65)	0.21	-6.25 (-43.70, 31.19)	0.74

HMO concentrations (nmol/mL)	PC1			PC2		
	β (95% CI)	P	P_BH_	β (95% CI)	P	P_BH_
2’FL	36.11 (-69.52, 141.74)	0.50	0.56	233.90 (111.96, 355.84)	3.0x10^-4^	**2.0x10^-3^**
3FL	24.32 (-11.11, 59.76)	0.18	0.33	-29.12 (-70.02, 11.79)	0.16	0.23
3’SL	17.81 (-6.41, 42.04)	0.15	0.31	-30.39 (-58.36, -2.43)	0.03	0.11
6’SL	4.20 (-6.67, 15.07)	0.44	0.56	25.41 (12.86, 37.96)	1.0x10^-4^	**0.002**
DFLac	5.44 (-5.64, 16.52)	0.33	0.45	-9.80 (-22.59, 2.99)	0.13	0.22
DFLNH	1.61 (-2.80, 6.03)	0.47	0.56	2.16 (-2.93, 7.26)	0.40	0.49
DFLNT	-22.90 (-52.82, 7.02)	0.13	0.31	-27.35 (-61.89, 7.19)	0.12	0.22
DSLNH	2.11 (-1.55, 5.77)	0.26	0.37	3.41 (-0.82, 7.64)	0.11	0.22
DSLNT	-3.38 (-9.17, 2.40)	0.25	0.37	-6.71 (-13.39, -0.03)	4.9x10^-2^	0.13
FDSLNH	2.67 (-0.63, 5.97)	0.11	0.31	2.93 (-0.88, 6.74)	0.13	0.22
FLNH	3.83 (-0.06, 7.71)	0.05	0.28	5.97 (1.48, 10.45)	0.01	**0.06**
LnNT	-11.52 (-23.39, 0.36)	0.06	0.28	1.15 (-12.56, 14.86)	0.87	0.87
LNT	-21.78 (-45.96, 2.41)	0.08	0.29	19.10 (-8.82, 47.02)	0.18	0.24
LNFP I	-11.48 (-50.44, 27.47)	0.56	0.59	-51.51 (-96.48, -6.53)	0.03	0.11
LNFP II	-13.68 (-27.91, 0.56)	0.06	0.28	12.31 (-4.13, 28.75)	0.14	0.22
LNFP III	0.68 (-0.42, 1.78)	0.22	0.37	-0.52 (-1.79, 0.75)	0.42	0.49
LNH	2.33 (0.07, 4.59)	0.04	0.28	0.99 (-1.62, 3.59)	0.45	0.51
LSTb	-0.30 (-2.04, 1.45)	0.74	0.74	-2.17 (-4.18, -0.15)	0.04	0.11
LSTc	-4.30 (-9.87, 1.27)	0.13	0.31	1.63 (-4.80, 8.06)	0.62	0.65

Multivariable linear regression analysis was used to examine the associations between PC1 and PC2 with HMO summary measures and HMO concentrations. Models adjusted for technical covariates (i.e., proportion of rRNA, volume of skim milk) as well as days postpartum, time of day of human milk collection, and number of breast feedings per day. Regressions where the outcome was HMO concentration were adjusted for multiple testing using the Benjamini-Hochberg procedure (BH). Bold indicates P_BH_ < 0.1.

^+^Diversity was estimated using Simpson’s Diversity measure.

In sensitivity analyses, we additionally adjusted for maternal BMI, maternal healthy eating index, and maternal age (**Supplementary Table 2**). Results from these analyses were largely similar to results from the main analyses, where the sum of all HMOs was positively associated with PC2 (P = 0.0004) and positively associated with HMO-bound fucose (P = 0.04). While not statistically significant (P = 0.09), the estimated association between PC2 and HMO diversity was negative. We also found that PC2 was positively associated with 2’FL and 6’SL.

### Individual EV-miRNAs are associated with HMO measures

As shown in **Table 4**, multivariable linear regression analysis, which adjusted for technical covariates, days postpartum, time of day of human milk collection, and number of breast feedings per day, revealed that 26 EV-miRNAs were associated with HMO diversity (all P < 0.05), 55 with the sum of all HMOs, 9 with HMO-bound sialic acid, and 33 with HMO-bound fucose. Complete results are shown in **Supplementary Table 3**. When examining individual HMO concentrations of 2’FL, 3FL, 3’SL, 6’SL, FLNH, LNFP I, and LNH, each were statistically significantly associated with levels of 7, 17, 1, 1, 4, 1, and 5 individual EV-miRNAs (respectively) after correction for multiple testing (P_BH_ < 0.1).

**Table 4:** Summary of the number of EV-miRNA associated with HMOs measures and concentrations via multivariable linear regression analysis.

HMO Summary Measures (nmol/mL)	P < 0.05 Count	P_BH_ < 0.10 Count
Diversity^+^	26	–
Sum of HMOs	55	–
HMO-bound sialic acid	9	–
HMO-bound fucose	33	–
HMO Concentrations (nmol/mL)	P < 0.05 Count	P_BH_ < 0.10 Count
2’FL	52	7
3’FL	63	17
3’SL	34	1
6’SL	52	1
DFLac	34	0
DFLNH	7	0
DFLNT	39	0
DSLNH	20	0
DSLNT	34	0
FDSLNH	26	0
FLNH	52	4
LnNT	21	0
LNT	29	0
LNFP I	29	1
LNFP II	22	0
LNFP III	10	0
LNH	58	5
LSTb	19	0
LSTc	13	0

Multivariable linear regression analysis was used to examine the associations between individual EV-miRNAs with HMO summary measures and HMO concentrations. Models adjusted for adjusted for technical covariates (i.e., proportion of rRNA, volume of skim milk) as well as days postpartum, human milk collection time, and breast feedings per day. “P < 0.05 Count” indicates the number of EV-miRNAs that were associated with each HMO measure based on P < 0.05. “P_BH_ Count” indicates the number of EV-miRNAs that were associated with each HMO measures based on P_BH_ < 0.10.

^+^Diversity was estimated using Simpson’s Diversity measure.

In sensitivity analyses where we additionally adjusted for maternal BMI, maternal healthy eating index (HEI), and maternal age, findings were largely consistent (**Supplementary Tables 4**, **5**). Among the 26 EV-miRNAs that were significantly associated with HMO diversity, 19 remained significantly associated after these additional adjustments. Among the 55 EV-miRNAs significantly associated with the sum of all HMOs in the main analysis, 49 remained significant in the sensitivity analysis. Among the 9 EV-miRNAs associated with HMO-bound sialic acid in the main analysis, 4 remained significant in the sensitivity analysis. Among the 33 EV-miRNAs which were associated with HMO-bound fucose in the main analysis, 27 of those remained significantly associated in the sensitivity analysis. Among the 7 EV-miRNAs associated with 2’FL concentration, 6 remained significant in the sensitivity analysis. Among the 17 EV-miRNAs which were associated with 3’FL concentration in the main analysis, 16 remained significant in the sensitivity analysis. Results for the 3’SL, 6’SL, and LNFP I were unchanged in sensitivity analyses. However, among the 4 EV-miRNAs associated with FLNH in the main analysis, only one EV-miRNA remained significant in the sensitivity analysis. Among the 5 EV-miRNAs associated with LNH in the main analysis, 4 remained significantly associated with LNH in the sensitivity analysis.

### Putative pathways of EV-miRNAs associated with HMO measures

Finally, we explored the functional pathways of predicted target genes for the EV-miRNAs that were associated with HMO expression. For example, we found that EV-miRNAs that were associated with HMO diversity, 2’FL, FLNH, LNFP I and LNH were also target genes involved in the Hippo signaling pathway (**Table 5**). EV-miRNAs associated with sum of HMOs, HMO-bound fucose, 3FL, 6’SL, FLNH, and LNH also target genes involved in the cell cycle pathway. Using the micro-T database, similar pathways were identified as being potential targets of EV-miRNAs that were associated with HMO measures (**Supplementary Table 6**).

**Table 5:** Top 5 most statistically significant putative pathways for miRNAs significantly associated with HMO characteristics.

HMO Summary Measures	Pathway	Merged P_FDR_
Diversity	Ubiquitin mediated proteolysis	1.2x10^-47^
	Pathways in cancer	8.7x10^-47^
	Hippo signaling pathway	1.5x10^-43^
	Colorectal cancer	8.7x10^-33^
	Proteoglycans in cancer	4.0x10^-31^
Sum of HMOs (nmol/mL)	Cell cycle	2.5x10^-104^
	Pathways in cancer	3.7x10^-102^
	p53 signaling pathway	2.1x10^-96^
	Proteoglycans in cancer	4.3x10^-92^
	FoxO signaling pathway	1.2x10^-84^
HMO-bound sialic acid (nmol/mL)	Focal adhesion	1.4x10^-8^
	p53 signaling pathway	1.4x10^-8^
	Colorectal cancer	5.3x10^-8^
	Autophagy-animal	1.5x10^-7^
	Small cell lung cancer	4.5x10^-7^
HMO-bound fucose (nmol/mL)	Ubiquitin mediated proteolysis	1.7x10^-63^
	Pathways in cancer	3.2x10^-63^
	p53 signaling pathway	3.0x10^-58^
	Proteoglycans in cancer	3.9x10^-49^
	Cell cycle	2.7x10^-47^
HMO Concentrations		
2’FL (nmol/mL)	Ubiquitin mediated proteolysis	7.5x10^-11^
	Hippo signaling pathway	5.9x10^-8^
	Colorectal cancer	4.2x10^-7^
	Pathways in cancer	6.7x10^-7^
	Oocyte meiosis	1.4x10^-6^
3’FL (nmol/mL)	Proteoglycans in cancer	4.4x10^-27^
	Cell cycle	2.7x10^-23^
	Pathways in cancer	5.1x10^-23^
	Hepatocellular carcinoma	2.5x10^-20^
	p53 signaling pathway	7.0x10^-19^
3’SL (nmol/mL)	Autophagy-animal	0.04
6’SL (nmol/mL)	Cell cycle	9.0x10^-10^
	Chronic myeloid leukemia	2.1x10^-5^
	FoxO signaling pathway	2.1x10^-5^
	Prostate cancer	6.5x10^-5^
	Colorectal cancer	8.1x10^-5^
FLNH (nmol/mL)	Hippo signaling pathway	2.9x10^-6^
	Salmonella infection	4.3x10^-5^
	Cell cycle	1.4x10^-4^
	Regulation of actin cytoskeleton	1.4x10^-4^
	Focal adhesion	4.9x10^-4^
LNFP I (nmol/mL)	Regulation of actin cytoskeleton	1.0x10^-3^
	Hippo signaling pathway	1.4x10^-3^
	RNA transport	2.1x10^-2^
	Proteoglycans in cancer	4.4x10^-2^
	MAPK signaling pathway	4.4x10^-2^
LNH (nmol/mL)	Pathways in cancer	4.9x10^-10^
	Proteoglycans in cancer	7.7x10^-8^
	Hippo signaling pathway	7.9x10^-8^
	FoxO signaling pathway	7.9x10^-8^
	Cell cycle	2.7x10^-7^

Putative pathways estimated using miRPath v4.0, KEGG pathway annotation, and TarBase v8.0 targets.

## Discussion

To our knowledge, this is the first study to explore the associations between EV-miRNAs and HMOs. We hypothesized that EV-miRNA levels in human milk may be associated with HMO concentrations and assessed this hypothesis using three distinct statistical approaches. We first utilized principal components as a data reduction technique to summarize EV-miRNA levels in human milk samples and found that PC2 was associated with HMO measures including HMO diversity, sum of HMOs, HMO-bound fucose, and concentrations of 2’FL, 6’SL, and FLNH. Next, we used multivariable linear regression to characterize the associations between individual EV-miRNA levels with HMO summary measures and individual HMO concentrations. We found that several EV-miRNA levels were associated with summary HMO measures including diversity, HMO-bound sialic acid, and HMO-bound fucose. We then used pathway analysis to explore the putative pathways of EV-miRNAs that were associated with HMOs. We identified several functional pathways through which EV-miRNAs may modulate HMO expression, including the cell cycle pathway and the hippo signaling pathway.

In this study, we found that PC2, which summarized levels of human milk EV-miRNAs, was associated with HMO summary measures and individual HMO concentrations. The highest loading scores for PC2 came from miR-183-5p and miR-151a-3p; miR-183-5p is highly expressed during lactation and is upregulated in mature human milk, compared to colostrum (56, 57). Levels of miR-183-5p have also been indicated in loss of breast epithelial cell polarity, which is associated with plasticity in early breast carcinoma (58). Additionally, miR-151a-3p may affect the growth hormone receptor (GHR), which controls human growth hormone (hGH), a key regulator of human lactation (39). Our study also identified several EV-miRNAs whose levels were associated with HMO summary measures and concentrations. For example, miR-30d-5p was negatively associated with HMO diversity and was also positively associated with sum of HMOs, HMO-bound fucose, and the concentration of 2’FL. miR-30d-5p is one of the most abundant human milk miRNAs (59) and has been shown to inhibit cell proliferation via cell cycle modulation in gallbladder (60) and pancreatic cancer (61). In our study, pathway analysis also indicated that miR-30d-5p is involved in pathways including cell cycle and hippo signaling. The hippo signaling pathway plays a key role in modulating cell proliferation (62, 63) and has previously been linked with maternal stress. For example, biological pathways related to hippo signaling were enriched among EV-miRNAs in mothers with high lifetime stress (including miR-30d-5p and miR-148a-3p) (63), suggesting that maternal stress may impact the composition of HMOs in milk via alterations in EV-miRNAs.

Our previous work in the Mother’s Milk Study has found that ambient air pollution exposure, including PM_2.5_ (i.e., particulate matter < 2.5 microns) and PM_10_, was associated with lower HMO diversity, and a higher sum of HMOs (64). We also found that greater exposure to PM was associated with higher 2’FL, 3FL, LNH, FLNH and lower HMO HMO-bound fucose, LNFP I, LNFP II, and DFLNT concentrations at 1-month postpartum. Our recent work in the same cohort also found that higher exposure to particulate matter during pregnancy (i.e., PM_2.5_ and PM_10_) was associated with several EV-miRNAs at 1-month postpartum; specifically, PM_10_ was positively associated with miR-200b-3p, miR-200c-3p and miR-125b. In the current study, we found that higher miR-200b-3p and miR-200c-3p levels were each associated with lower HMO diversity. Though not statistically significant after adjustment for multiple testing, we also saw that higher levels of miR-200b-3p and miR-200c-3p were associated with higher 2’FL, and lower DFLNT, LNnT, and LNFP I. We also found that miR-200c-3p was positively associated with sum of HMOs and HMO-bound fucose. Lastly, higher levels of miR-125b-5p were associated with lower HMO diversity and higher levels of the sum of HMOs, HMO-bound fucose, and 2’FL. Though not statistically significant after adjustment for multiple testing, we saw that levels of miR-125b-5p were associated with lower DFLNT and LNFP I and higher FLNH. Collectively, this body of work suggests a potential link between environmental exposures and HMO concentrations that may be mediated by specific EV-miRNAs; further studies should therefore continue to consider environmental impacts on gene expression and HMO regulation.

The present study is novel in its investigation of the relationships between levels of EV-miRNAs and HMO concentrations in human milk. However, this study also has important limitations worth noting. Firstly, this cross-sectional study was conducted on samples collected at 1-month postpartum and therefore causality and temporality of the associations cannot be assessed. However, this study can provide hypothesis generation for future, longitudinal studies. Additionally, the original aim of this study was to assess human milk for HMOs. Thus, milk was frozen for storage, which could have resulted in contamination from lysed cells or significant loss of milk EVs (65). However, a representative subset of samples utilized in this analysis underwent additional testing and were determined to be positive for eight EV markers and showed minimal contamination by GM130, a Golgi matrix protein which can be a marker for cellular contamination (40). Lastly, our sample was fully Latino and was largely comprised of mothers with overweight and obesity and who intended to breastfeed for at least 6 months. We additionally excluded participants who were classified as non-secretors. Each of these factors may limit the generalizability of our findings.

## Conclusions

This study provides preliminary evidence that EV-miRNA levels may influence HMO summary measures and concentrations in human milk at 1-month postpartum. While further analyses are needed, this work contributes to the growing literature characterizing maternal factors that impact HMO biosynthesis. Understanding these influences is crucial, as HMOs play a significant role in shaping the infant gut microbiome, supporting immune development, and protecting against infections, thereby impacting infant health and development.

## Data Availability

The datasets presented in this article are not readily available because they include potentially identifying information on human subjects. Requests to access the datasets should be directed to taldere1@jhu.edu.

## References

[1] Gillman MW Rifas-Shiman SL Camargo CA Jr. Berkey CS Frazier AL Rockett HR. Risk of overweight among adolescents who were breastfed as infants. JAMA. (2001) 285:2461–7. doi: 10.1001/jama.285.19.2461 11368698

[2] Bider-Canfield Z Martinez MP Wang X Yu W Bautista MP Brookey J. Maternal obesity, gestational diabetes, breastfeeding and childhood overweight at age 2 years. Pediatr Obes. (2017) 12:171–8. doi: 10.1111/ijpo.12125 26956226

[3] Belfort MB Rifas-Shiman SL Kleinman KP Guthrie LB Bellinger DC Taveras EM. Infant feeding and childhood cognition at ages 3 and 7 years: Effects of breastfeeding duration and exclusivity. JAMA Pediatr. (2013) 167:836–44. doi: 10.1001/jamapediatrics.2013.455 PMC3998659 23896931

[4] Giugliani ERJ Horta BL Loret de Mola C Lisboa BO Victora CG. Effect of breastfeeding promotion interventions on child growth: a systematic review and meta-analysis. Acta Paediatr. (2015) 104:20–9. doi: 10.1111/apa.13160 26361071

[5] Horta BL Loret de Mola C Victora CG. Breastfeeding and intelligence: a systematic review and meta-analysis. Acta Paediatr. (2015) 104:14–9. doi: 10.1111/apa.13139 26211556

[6] Kramer MS Guo T Platt RW Sevkovskaya Z Dzikovich I Collet JP. Infant growth and health outcomes associated with 3 compared with 6 mo of exclusive breastfeeding. Am J Clin Nutr. (2003) 78:291–5. doi: 10.1093/ajcn/78.2.291 12885711

[7] Horta BL Loret de Mola C Victora CG. Long-term consequences of breastfeeding on cholesterol, obesity, systolic blood pressure and type 2 diabetes: a systematic review and meta-analysis. Acta Paediatrica. (2015) 104:30–7. doi: 10.1111/apa.13133 26192560

[8] Lodge C Tan D Lau M Dai X Tham R Lowe AJ. Breastfeeding and asthma and allergies: a systematic review and meta-analysis. Acta Paediatr. (2015) 104:38–53. doi: 10.1111/apa.13132 26192405

[9] Bode L. Human milk oligosaccharides: Every baby needs a sugar mama. Glycobiology. (2012) 22:1147–62. doi: 10.1093/glycob/cws074 PMC3406618 22513036

[10] McGuire MK Meehan CL McGuire MA Williams JE Foster J Sellen DW. What’s normal? Oligosaccharide concentrations and profiles in milk produced by healthy women vary geographically. Am J Clin Nutr. (2017) 105:1086–100. doi: 10.3945/ajcn.116.139980 PMC5402033 28356278

[11] Rosa F Sharma AK Gurung M Casero D Matazel K Bode L. Human milk oligosaccharides impact cellular and inflammatory gene expression and immune response. Front Immunol. (2022) 13:907529. doi: 10.3389/fimmu.2022.907529 35844612 PMC9278088

[12] Asakuma S Hatakeyama E Urashima T Ashida H Hirose JM Kitaoka M. Physiology of consumption of human milk oligosaccharides by infant gut-associated bifidobacteria *. J Biol Chem. (2011) 286:34583–92. doi: 10.1074/jbc.M111.248138 PMC3186357 21832085

[13] Marcobal A Barboza M Froehlich JW Block DE German JB Lebrilla CB. Consumption of human milk oligosaccharides by gut-related microbes. J Agric Food Chem. (2010) 58:5334–40. doi: 10.1021/jf9044205 PMC2866150 20394371

[14] LoCascio RG Ninonuevo MR Freeman SL Sela DA Grimm R Lebrilla CB. Glycoprofiling of bifidobacterial consumption of human milk oligosaccharides demonstrates strain specific, preferential consumption of small chain glycans secreted in early human lactation. J Agric Food Chem. (2007) 55:8914–9. doi: 10.1021/jf0710480 17915960

[15] Newburg DS Ruiz-Palacios GM Morrow AL. Human milk glycans protect infants against enteric pathogens. Annu Rev Nutr. (2005) 25:37–58. doi: 10.1146/annurev.nutr.25.050304.092553 16011458

[16] Kunz C Rudloff S Baier W Klein N Strobel S. Oligosaccharides in human milk: structural, functional, and metabolic aspects. Annu Rev Nutr. (2000) 20:699–722. doi: 10.1146/annurev.nutr.20.1.699 10940350

[17] Kuntz S Rudloff S Kunz C. Oligosaccharides from human milk influence growth-related characteristics of intestinally transformed and non-transformed intestinal cells. Br J Nutr. (2008) 99:462–71. doi: 10.1017/S0007114507824068 17925055

[18] Eiwegger T Stahl B Haidl P Schmitt J Boehm G Dehlink E. Prebiotic oligosaccharides: *In vitro* evidence for gastrointestinal epithelial transfer and immunomodulatory properties. Pediatr Allergy Immunol. (2010) 21:1179–88. doi: 10.1111/j.1399-3038.2010.01062.x 20444147

[19] Wang B. Sialic acid is an essential nutrient for brain development and cognition. Annu Rev Nutr. (2009) 29:177–222. doi: 10.1146/annurev.nutr.28.061807.155515 19575597

[20] Lagström H Rautava S Ollila H Kaljonen A Turta O Mäkelä J. Associations between human milk oligosaccharides and growth in infancy and early childhood. Am J Clin Nutr. (2020) 111:769–78. doi: 10.1093/ajcn/nqaa010 PMC7138667 32068776

[21] Williams JE McGuire MK Meehan CL McGuire MA Brooker SL Kamau-Mbuthia EW. Key genetic variants associated with variation of milk oligosaccharides from diverse human populations. Genomics. (2021) 113:1867–75. doi: 10.1016/j.ygeno.2021.04.004 33831438

[22] Thurl S Munzert M Henker J Boehm G Müller-Werner B Jelinek J. Variation of human milk oligosaccharides in relation to milk groups and lactational periods. Br J Nutr. (2010) 104:1261–71. doi: 10.1017/S0007114510002072 20522272

[23] Plows JF Berger PK Jones RB Alderete TL Yonemitsu C Najera JA. Longitudinal changes in human milk oligosaccharides (HMOs) over the course of 24 months of lactation. J Nutr. (2021) 151:876–82. doi: 10.1093/jn/nxaa427 PMC8030713 33693851

[24] Han SM Derraik JGB Binia A Sprenger N Vickers MH Cutfield WS. Maternal and infant factors influencing human milk oligosaccharide composition: beyond maternal genetics. J Nutr. (2021) 151:1383–93. doi: 10.1093/jn/nxab028 33768224

[25] Siziba LP Mank M Stahl B Gonsalves J Blijenberg B Rothenbacher D. Human milk oligosaccharide profiles over 12 months of lactation: the ulm SPATZ health study. Nutrients. (2021) 13:1973. doi: 10.3390/nu13061973 34201331 PMC8228739

[26] Thum C Wall CR Weiss GA Wang W Szeto IMY Day L. Changes in HMO concentrations throughout lactation: influencing factors, health effects and opportunities. Nutrients. (2021) 13:2272. doi: 10.3390/nu13072272 34209241 PMC8308359

[27] Azad MB Robertson B Atakora F Becker AB Subbarao P Moraes TJ. Human milk oligosaccharide concentrations are associated with multiple fixed and modifiable maternal characteristics, environmental factors, and feeding practices. J Nutr. (2018) 148:1733–42. doi: 10.1093/jn/nxy175 30247646

[28] Samuel TM Binia A de Castro CA Thakkar SK Billeaud C Agosti M. Impact of maternal characteristics on human milk oligosaccharide composition over the first 4 months of lactation in a cohort of healthy European mothers. Sci Rep. (2019) 9:11767. doi: 10.1038/s41598-019-48337-4 31409852 PMC6692355

[29] Saben JL Sims CR Abraham A Bode L Andres A. Human milk oligosaccharide concentrations and infant intakes are associated with maternal overweight and obesity and predict infant growth. Nutrients. (2021) 13:446. doi: 10.3390/nu13020446 33572881 PMC7911788

[30] Carr LE Virmani MD Rosa F Munblit D Matazel KS Elolimy AA. Role of human milk bioactives on infants’ Gut and immune health. Front Immunol. (2021) 12:604080. doi: 10.3389/fimmu.2021.604080 33643310 PMC7909314

[31] Alsaweed M Lai CT Hartmann PE Geddes DT Kakulas F. Human milk miRNAs primarily originate from the mammary gland resulting in unique miRNA profiles of fractionated milk. Sci Rep. (2016) 6:20680. doi: 10.1038/srep20680 26854194 PMC4745068

[32] Tanaka T Haneda S Imakawa K Sakai S Nagaoka K. A microRNA, miR-101a, controls mammary gland development by regulating cyclooxygenase-2 expression. Differentiation. (2009) 77:181–7. doi: 10.1016/j.diff.2008.10.001 19281778

[33] Zhou Q Li M Wang X Li Q Wang T Zhu Q. Immune-related microRNAs are abundant in breast milk exosomes. Int J Biol Sci. (2012) 8:118–23. doi: 10.7150/ijbs.8.118 PMC3248653 22211110

[34] Baier SR Nguyen C Xie F Wood JR Zempleni J. MicroRNAs are absorbed in biologically meaningful amounts from nutritionally relevant doses of cow milk and affect gene expression in peripheral blood mononuclear cells, HEK-293 kidney cell cultures, and mouse livers. J Nutr. (2014) 144:1495–500. doi: 10.3945/jn.114.196436 PMC4162473 25122645

[35] Kahn S Liao Y Du X Xu W Li J Lönnerdal B. Exosomal microRNAs in milk from mothers delivering preterm infants survive *in vitro* digestion and are taken up by human intestinal cells. Mol Nutr Food Res. (2018) 62:e1701050. doi: 10.1002/mnfr.201701050 29644801

[36] Liao Y Du X Li J Lönnerdal B. Human milk exosomes and their microRNAs survive digestion *in vitro* and are taken up by human intestinal cells. Mol Nutr Food Res. (2017) 61:1700082. doi: 10.1002/mnfr.201700082 28688106

[37] Carney MC Tarasiuk A DiAngelo SL Silveyra P Podany A Birch LL. Metabolism-related microRNAs in maternal breast milk are influenced by premature delivery. Pediatr Res. (2017) 82:226–36. doi: 10.1038/pr.2017.54 PMC5552431 28422941

[38] Xi Y Jiang X Li R Chen M Song W Li X. The levels of human milk microRNAs and their association with maternal weight characteristics. Eur J Clin Nutr. (2016) 70:445–9. doi: 10.1038/ejcn.2015.168 26486303

[39] Alsaweed M Lai CT Hartmann PE Geddes DT Kakulas F. Human milk cells contain numerous miRNAs that may change with milk removal and regulate multiple physiological processes. Int J Mol Sci. (2016) 17:956. doi: 10.3390/ijms17060956 27322254 PMC4926489

[40] Holzhausen EA Kupsco A Chalifour BN Patterson WB Schmidt KA Mokhtari P. Influence of technical and maternal-infant factors on the measurement and expression of extracellular miRNA in human milk. Front Immunol. (2023) 14:1151870. doi: 10.3389/fimmu.2023.1151870 37492577 PMC10363855

[41] Holzhausen EA Kupsco A Chalifour BN Patterson WB Schmidt KA Mokhtari P. Human milk EV-miRNAs: a novel biomarker for air pollution exposure during pregnancy. Environ Res: Health. (2023) 1:035002. doi: 10.1088/2752-5309/ace075 37692372 PMC10486183

[42] Wild LE Alderete TL Naik NC Patterson WB Berger PK Jones RB. Specific amino acids but not total protein attenuate postpartum weight gain among Hispanic women from Southern California. Food Sci Nutr. (2021) 9:1842–50. doi: 10.1002/fsn3.2085 PMC8020954 33841803

[43] Krebs-Smith SM Pannucci TE Subar AF Tooze JA Wilson MM Reedy J. Update of the healthy eating index: HEI-2015. J Acad Nutr Dietetics. (2018) 118:1591–602. doi: 10.1016/j.jand.2018.05.021 PMC6719291 30146071

[44] Patterson WB Glasson J Naik N Jones RB Berger PK Plows JF. Prenatal exposure to ambient air pollutants and early infant growth and adiposity in the Southern California Mother’s Milk Study. Environ Health. (2021) 20:67. doi: 10.1186/s12940-021-00753-8 34090448 PMC8180163

[45] Fields DA Demerath EW. Relationship of insulin, glucose, leptin, IL-6 and TNF-α in human breast milk with infant growth and body composition. Pediatr Obes. (2012) 7:304–12. doi: 10.1111/j.2047-6310.2012.00059.x PMC3393795 22577092

[46] Hahn W Kim J Song S Park S Kang NM. The human milk oligosaccharides are not affected by pasteurization and freeze-drying. J Maternal-Fetal Neonatal Med. (2019) 32:985–91. doi: 10.1080/14767058.2017.1397122 29108433

[47] Kupsco A Prada D Valvi D Hu L Petersen MS Boull B. Human milk extracellular vesicle miRNA expression and associations with maternal characteristics in a population-based cohort from the Faroe Islands. Sci Rep. (2021) 11:5840. doi: 10.1038/s41598-021-84809-2 33712635 PMC7970999

[48] Van Deun J Mestdagh P Agostinis P Akay O Anand S Anckaert J. EV-TRACK: transparent reporting and centralizing knowledge in extracellular vesicle research. Nat Methods. (2017) 14:228–32. doi: 10.1038/nmeth.4185 28245209

[49] Rozowsky J Kitchen RR Park JJ Subramanian SL Milosavljevic A Gerstein M. exceRpt: A comprehensive analytic platform for extracellular RNA profiling. Cell Syst. (2019) 8:352–357.e3. doi: 10.1016/j.cels.2019.03.004 30956140 PMC7079576

[50] Robinson MD McCarthy DJ Smyth GK. edgeR: a Bioconductor package for differential expression analysis of digital gene expression data. Bioinformatics. (2010) 26:139–40. doi: 10.1093/bioinformatics/btp616 PMC2796818 19910308

[51] Benjamini Y Hochberg Y. Controlling the false discovery rate: A practical and powerful approach to multiple testing. J R Stat Soc Ser B Stat Metholol. (1995) 57:289–300. doi: 10.1111/j.2517-6161.1995.tb02031.x

[52] Tastsoglou S Skoufos G Miliotis M Karagkouni D Koutsoukos I Karavangeli A. DIANA-miRPath v4.0: expanding target-based miRNA functional analysis in cell-type and tissue contexts. Nucleic Acids Res. (2023) 51:W154–9. doi: 10.1093/nar/gkad431 PMC10320185 37260078

[53] Karagkouni D Paraskevopoulou MD Chatzopoulos S Vlachos IS Tastsoglou S Kanellos I. DIANA-TarBase v8: a decade-long collection of experimentally supported miRNA–gene interactions. Nucleic Acids Res. (2018) 46:D239–45. doi: 10.1093/nar/gkx1141 PMC5753203 29156006

[54] Paraskevopoulou MD Georgakilas G Kostoulas N Vlachos IS Vergoulis T Reczko M. DIANA-microT web server v5.0: service integration into miRNA functional analysis workflows. Nucleic Acids Res. (2013) 41:W169–73. doi: 10.1093/nar/gkt393 PMC3692048 23680784

[55] Simpson MR Brede G Johansen J Johnsen R Storrø O Sætrom P. Human breast milk miRNA, maternal probiotic supplementation and atopic dermatitis in offspring. PloS One. (2015) 10:e0143496. doi: 10.1371/journal.pone.0143496 26657066 PMC4682386

[56] Wu F Zhi Z Zu R Liang Z Wang F Li X. Exploration of microRNA profiles in human colostrum. Ann Trans Med. (2020) 8:1170–0. doi: 10.21037/atm-20-5709 PMC7576086 33241019

[57] Li Z Liu H Jin X Lo L Liu J. Expression profiles of microRNAs from lactating and non-lactating bovine mammary glands and identification of miRNA related to lactation. BMC Genomics. (2012) 13:731. doi: 10.1186/1471-2164-13-731 23270386 PMC3551688

[58] Naser Al Deen N Atallah Lanman N Chittiboyina S Fostok S Nasr R Lelièvre S. Over-expression of miR-183-5p or miR-492 triggers invasion and proliferation and loss of polarity in non-neoplastic breast epithelium. Sci Rep. (2022) 12:21974. doi: 10.1038/s41598-022-25663-8 36539576 PMC9768134

[59] Tingö L Ahlberg E Johansson L Pedersen SA Chawla K Sætrom P. Non-coding RNAs in human breast milk: A systematic review. Front Immunol. (2021) 12:725323. doi: 10.3389/fimmu.2021.725323 34539664 PMC8440964

[60] Ye YY Mei JW Xiang SS Li HF Ma Q Song XL. MicroRNA-30a-5p inhibits gallbladder cancer cell proliferation, migration and metastasis by targeting E2F7. Cell Death Dis. (2018) 9:1–12. doi: 10.1038/s41419-018-0444-x 29540696 PMC5852001

[61] Xu X Zong K Wang X Dou D Lv P Zhang Z. miR-30d suppresses proliferation and invasiveness of pancreatic cancer by targeting the SOX4/PI3K-AKT axis and predicts poor outcome. Cell Death Dis. (2021) 12:1–14. doi: 10.1038/s41419-021-03576-0 33824274 PMC8024348

[62] Meng Z Moroishi T Guan KL. Mechanisms of Hippo pathway regulation. Genes Dev. (2016) 30:1–17. doi: 10.1101/gad.274027.115 26728553 PMC4701972

[63] Bozack AK Colicino E Rodosthenous R Bloomquist TR Baccarelli AA Wright RO. Associations between maternal lifetime stressors and negative events in pregnancy and breast milk-derived extracellular vesicle microRNAs in the programming of intergenerational stress mechanisms (PRISM) pregnancy cohort. Epigenetics. (2021) 16:389–404. doi: 10.1080/15592294.2020.1805677 32777999 PMC7996083

[64] Naik NC Holzhausen EA Chalifour BN Coffman MM Lurmann F Goran MI. Air pollution exposure may impact the composition of human milk oligosaccharides. Sci Rep. (2024) 14:6730. doi: 10.1038/s41598-024-57158-z 38509153 PMC10954706

[65] Zonneveld MI Brisson AR van Herwijnen MJC Tan S van de Lest CHA Redegeld FA. Recovery of extracellular vesicles from human breast milk is influenced by sample collection and vesicle isolation procedures. J Extracellular Vesicles. (2014) 3:24215. doi: 10.3402/jev.v3.24215 PMC4139932 25206958

