# Supplementary material for: Associations between human milk EV-miRNAs and oligosaccharide concentrations in human milk
Source: Front Immunol. 2024 Nov 20;15:1463463. doi: 10.3389/fimmu.2024.1463463 (PMC11614774; doi:10.3389/fimmu.2024.1463463)

**Supplemental Figure 1.** Principal components plot of PC1 and PC2, summarizing EV-miRNA levels.**
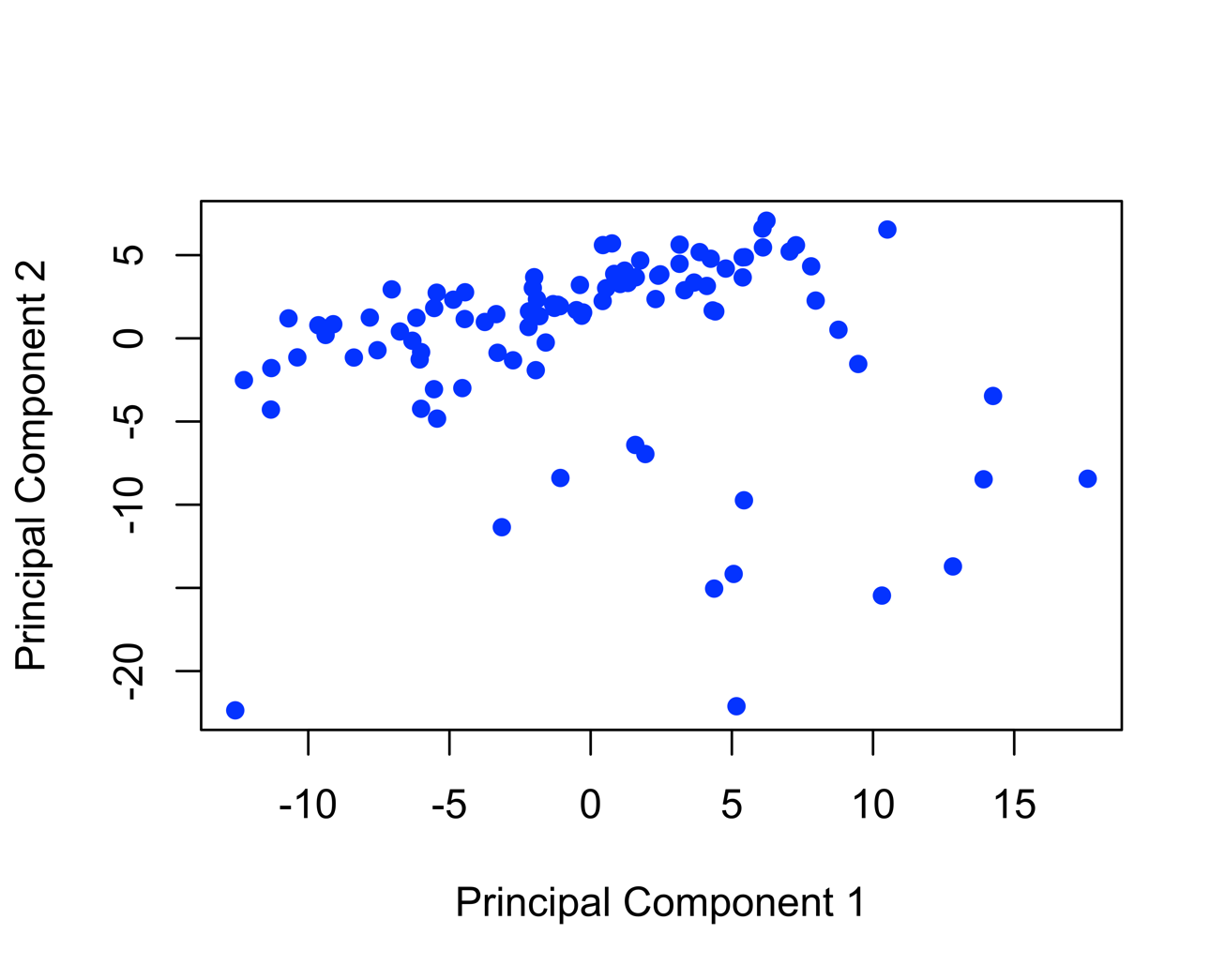
**

**Supplemental Figure 2.** Plot enumerating the proportion of variance explained by principal components 1 through 10.


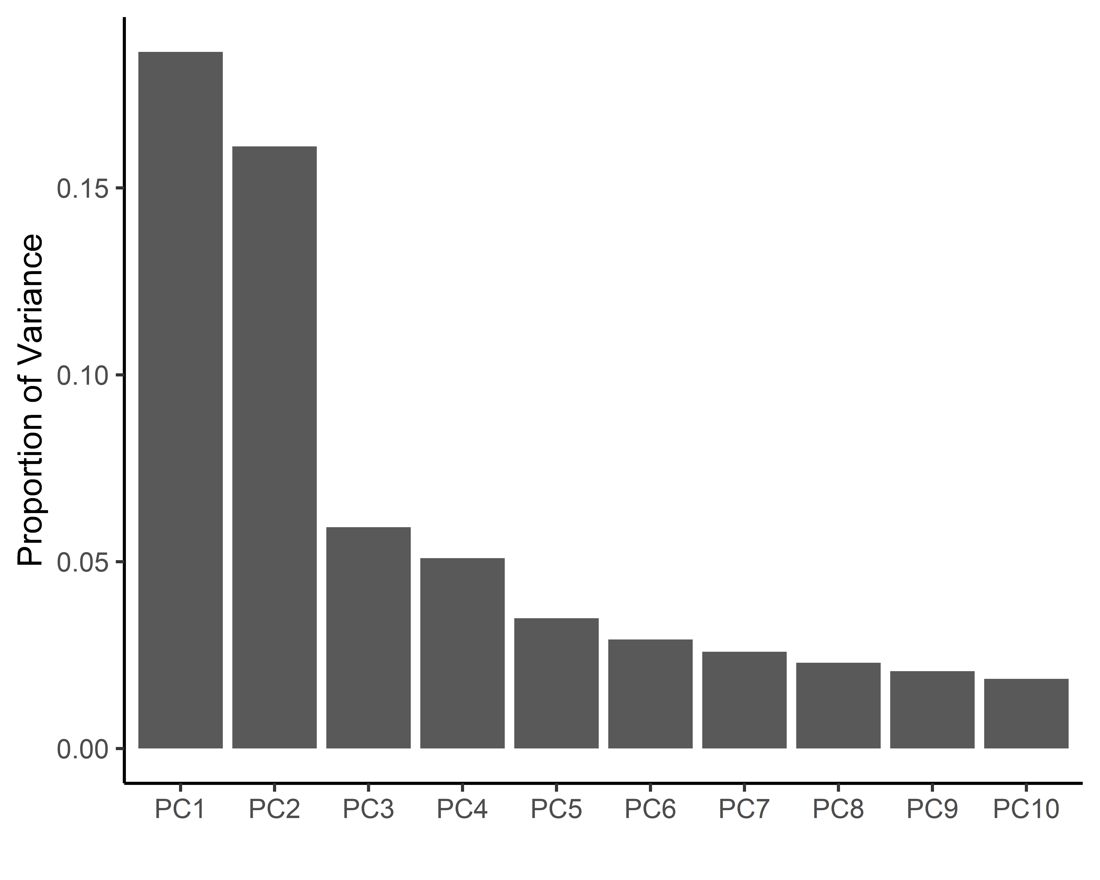

Supplement: Supplementary file 1 [file DataSheet1.docx]
