# Supplementary material for: Associations between human milk EV-miRNAs and oligosaccharide concentrations in human milk
Source: Front Immunol. 2024 Nov 20;15:1463463. doi: 10.3389/fimmu.2024.1463463 (PMC11614774; doi:10.3389/fimmu.2024.1463463)
Supplement: Supplementary file 2 [file Table1.docx]

**Supplemental Table 1.** EV-miRNA loading scores for principal components 1 and 2.

| **PC1** | | **PC2** | |
| --- | --- | --- | --- |
| **EV-miRNA** | **Loading Score** | **EV-miRNA** | **Loading Score** |
| miR-423-5p | 0.12 | miR-183-5p | 0.13 |
| miR-320a | 0.12 | miR-151a-3p | 0.13 |
| miR-342-5p | 0.11 | miR-511-5p | 0.12 |
| let-7b-5p | 0.11 | miR-99b-5p | 0.11 |
| miR-193a-5p | 0.11 | miR-3615 | 0.11 |
| miR-148b-3p | -0.15 | miR-21-5p | -0.15 |
| miR-30e-5p | -0.14 | miR-500a-3p | -0.15 |
| miR-30a-5p | -0.14 | miR-146a-5p | -0.14 |
| miR-141-3p | -0.13 | miR-629-5p | -0.14 |
| miR-30b-5p | -0.13 | miR-502-3p | -0.13 |

**Supplemental Table 1.** Shows the loading scores for the EV-miRNAs that contributed most to PC1 and PC2 (top 5 highest and lowest loading scores). A higher magnitude indicates a greater contribution to the PC.
