# Supplementary material for: Associations between human milk EV-miRNAs and oligosaccharide concentrations in human milk
Source: Front Immunol. 2024 Nov 20;15:1463463. doi: 10.3389/fimmu.2024.1463463 (PMC11614774; doi:10.3389/fimmu.2024.1463463)
Supplement: Supplementary file 3 [file Table2.docx]

**Supplemental Table 2.** PC2 was associated with several HMO measures after additionally adjusting for maternal body mass index, maternal healthy eating index, and mother age.

| **HMO Summary Measures (nmol/mL)** | | **PC1** | | | | | **PC2** | | | |
| --- | --- | --- | --- | --- | --- | --- | --- | --- | --- | --- |
|  |  | **β (95% CI)** | | **P** | | | **β (95% CI)** | | **P** | |
| Diversity^+^ | | -0.02 (-0.09, 0.05) | | 0.54 | | | -0.07 (-0.16, 0.01) | | 0.09 | |
| Sum of HMOs | | 4.78 (-59.11, 68.66) | | 0.88 | | | 136.71 (62.72, 210.70) | | 0.0004 | |
| HMO-bound fucose | | 6.38 (-69.94, 82.71) | | 0.87 | | | 94.02 (5.62, 182.42) | | 0.04 | |
| HMO-bound sialic acid | | 15.88 (-16.85, 48.61) | | 0.34 | | | -3.61 (-41.52, 34.29) | | 0.85 | |
| **HMO concentrations (nmol/mL)** | **PC1** | | | | | **PC2** | | | | |
|  | **β (95% CI)** | | **P** | | **P_BH_** | **β (95% CI)** | | **P** | | **P_BH_** |
| 2’FL | 29.49 (-77.02, 136.00) | | 0.58 | | 0.65 | 210.85 (87.48, 334.21) | | 0.001 | | 0.01 |
| 3FL | 26.10 (-9.99, 62.19) | | 0.15 | | 0.33 | -33.78 (-75.58, 8.01) | | 0.11 | | 0.20 |
| 3’SL | 14.55 (-10.00, 39.09) | | 0.24 | | 0.46 | -30.22 (-58.65, -1.80) | | 0.04 | | 0.15 |
| 6’SL | 3.38 (-7.78, 14.55) | | 0.55 | | 0.65 | 24.42 (11.48, 37.35) | | 0.0003 | | 0.006 |
| DFLac | 4.32 (-7.05, 15.68) | | 0.45 | | 0.57 | -10.51 (-23.67, 2.65) | | 0.12 | | 0.20 |
| DFLNH | 2.14 (-2.26, 6.55) | | 0.34 | | 0.49 | 3.33 (-1.78, 8.43) | | 0.20 | | 0.27 |
| DFLNT | -23.71 (-54.04, 6.61) | | 0.12 | | 0.33 | -22.09 (-57.21, 13.03) | | 0.21 | | 0.27 |
| DSLNH | 1.84 (-1.82, 5.50) | | 0.32 | | 0.49 | 4.17 (-0.07, 8.41) | | 0.05 | | 0.15 |
| DSLNT | -3.29 (-9.20, 2.61) | | 0.27 | | 0.47 | -6.12 (-12.96, 0.73) | | 0.08 | | 0.19 |
| FDSLNH | 2.70 (-0.70, 6.10) | | 0.12 | | 0.33 | 3.20 (-0.73, 7.14) | | 0.11 | | 0.20 |
| FLNH | 3.90 (0.04, 7.77) | | 0.048 | | 0.32 | 5.01 (0.53, 9.49) | | 0.03 | | 0.15 |
| LnNT | -11.30 (-23.51, 0.92) | | 0.07 | | 0.32 | 2.32 (-11.83, 16.46) | | 0.75 | | 0.75 |
| LNT | -21.81 (-46.73, 3.11) | | 0.09 | | 0.32 | 18.28 (-11.58, 47.14) | | 0.21 | | 0.27 |
| LNFP I | -7.90 (-47.42, 31.63) | | 0.69 | | 0.72 | -45.18 (-90.95, 0.59) | | 0.05 | | 0.15 |
| LNFP II | -13.89 (-28.50, 0.78) | | 0.06 | | 0.32 | 13.00 (-3.96, 29.96) | | 0.13 | | 0.21 |
| LNFP III | 0.46 (-0.64, 1.55) | | 0.41 | | 0.55 | -0.52 (-1.78, 0.74) | | 0.42 | | 0.49 |
| LNH | 2.31 (-0.01, 4.64) | | 0.05 | | 0.32 | 0.89 (-1.80, 3.58) | | 0.51 | | 0.57 |
| LSTb | -0.33 (-2.13, 1.48) | | 0.72 | | 0.72 | -2.15 (-4.24, -0.06) | | 0.04 | | 0.15 |
| LSTc | -4.22 (-9.97, 1.54) | | 0.15 | | 0.33 | 1.83 (-4.83, 8.49) | | 0.59 | | 0.62 |

**Supplemental Table 2.** Multivariable linear regression analysis was used to examine the associations between PC1 and PC2 with HMO summary measures and HMO concentrations. Models adjusted for technical covariates (i.e., proportion of rRNA, volume of skim milk) as well as days postpartum, human milk collection time, breast feedings per day, maternal body mass index, maternal healthy eating index, and mother age.

^+^Diversity was estimated using Simpson’s Diversity measure.
