# Supplementary material for: Associations between human milk EV-miRNAs and oligosaccharide concentrations in human milk
Source: Front Immunol. 2024 Nov 20;15:1463463. doi: 10.3389/fimmu.2024.1463463 (PMC11614774; doi:10.3389/fimmu.2024.1463463)
Supplement: Supplementary file 5 [file Table4.docx]

**Supplemental Figure 1.** Principal components plot of PC1 and PC2, summarizing EV-miRNA levels.**
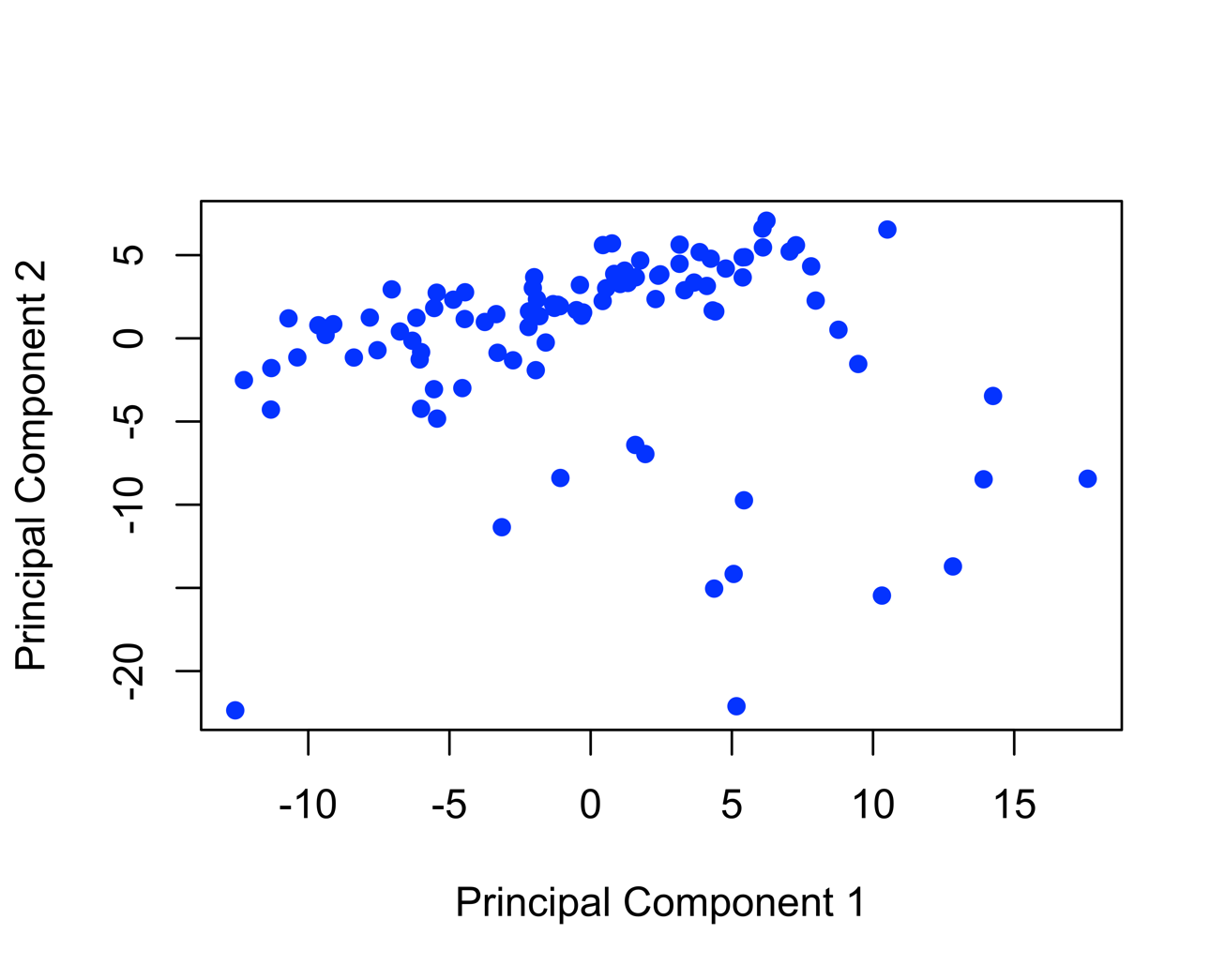
**

**Supplemental Figure 2.** Plot enumerating the proportion of variance explained by principal components 1 through 10.


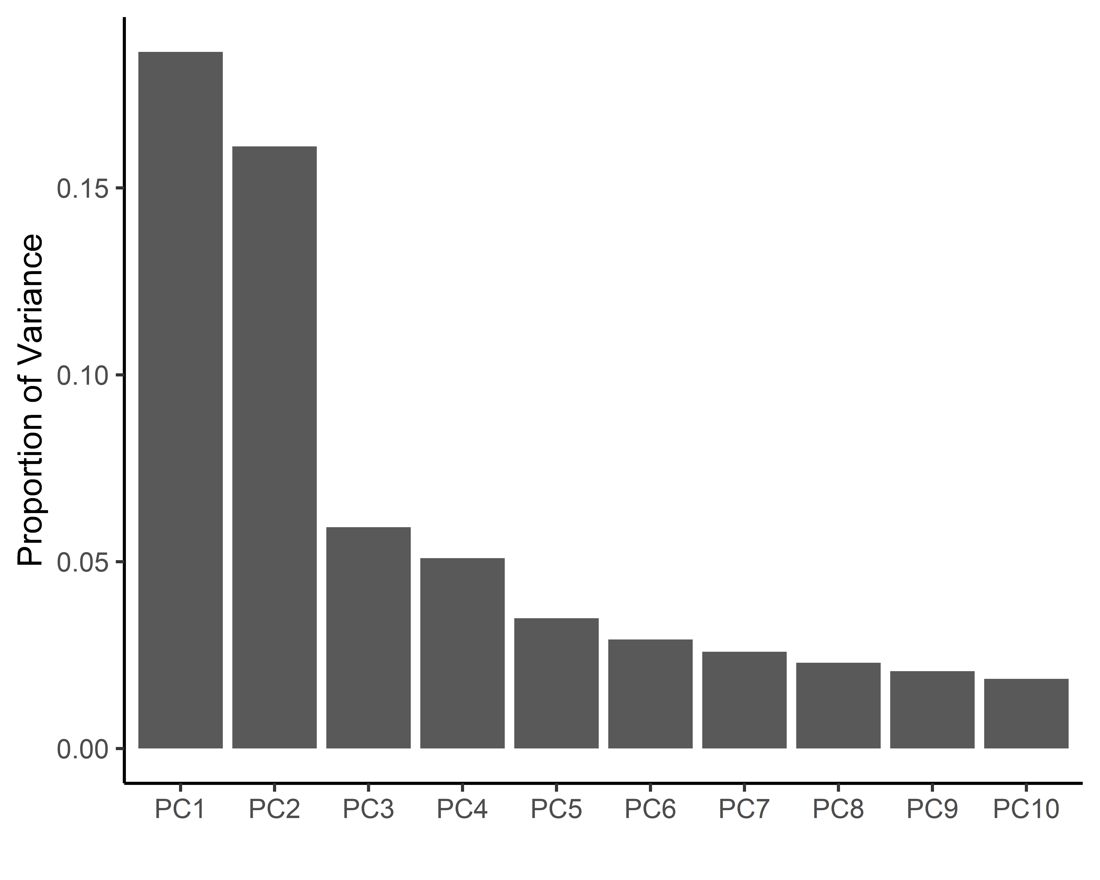


**Supplemental Table 1.** EV-miRNA loading scores for principal components 1 and 2.

| **PC1** | | **PC2** | |
| --- | --- | --- | --- |
| **EV-miRNA** | **Loading Score** | **EV-miRNA** | **Loading Score** |
| miR-423-5p | 0.12 | miR-183-5p | 0.13 |
| miR-320a | 0.12 | miR-151a-3p | 0.13 |
| miR-342-5p | 0.11 | miR-511-5p | 0.12 |
| let-7b-5p | 0.11 | miR-99b-5p | 0.11 |
| miR-193a-5p | 0.11 | miR-3615 | 0.11 |
| miR-148b-3p | -0.15 | miR-21-5p | -0.15 |
| miR-30e-5p | -0.14 | miR-500a-3p | -0.15 |
| miR-30a-5p | -0.14 | miR-146a-5p | -0.14 |
| miR-141-3p | -0.13 | miR-629-5p | -0.14 |
| miR-30b-5p | -0.13 | miR-502-3p | -0.13 |

**Supplemental Table 1.** Shows the loading scores for the EV-miRNAs that contributed most to PC1 and PC2 (top 5 highest and lowest loading scores). A higher magnitude indicates a greater contribution to the PC.

**Supplemental Table 2.** PC2 was associated with several HMO measures after additionally adjusting for maternal body mass index, maternal healthy eating index, and mother age.

| **HMO Summary Measures (nmol/mL)** | | **PC1** | | | | | **PC2** | | | |
| --- | --- | --- | --- | --- | --- | --- | --- | --- | --- | --- |
|  |  | **β (95% CI)** | | **P** | | | **β (95% CI)** | | **P** | |
| Diversity^+^ | | -0.02 (-0.09, 0.05) | | 0.54 | | | -0.07 (-0.16, 0.01) | | 0.09 | |
| Sum of HMOs | | 4.78 (-59.11, 68.66) | | 0.88 | | | 136.71 (62.72, 210.70) | | 0.0004 | |
| HMO-bound fucose | | 6.38 (-69.94, 82.71) | | 0.87 | | | 94.02 (5.62, 182.42) | | 0.04 | |
| HMO-bound sialic acid | | 15.88 (-16.85, 48.61) | | 0.34 | | | -3.61 (-41.52, 34.29) | | 0.85 | |
| **HMO concentrations (nmol/mL)** | **PC1** | | | | | **PC2** | | | | |
|  | **β (95% CI)** | | **P** | | **P_BH_** | **β (95% CI)** | | **P** | | **P_BH_** |
| 2’FL | 29.49 (-77.02, 136.00) | | 0.58 | | 0.65 | 210.85 (87.48, 334.21) | | 0.001 | | 0.01 |
| 3FL | 26.10 (-9.99, 62.19) | | 0.15 | | 0.33 | -33.78 (-75.58, 8.01) | | 0.11 | | 0.20 |
| 3’SL | 14.55 (-10.00, 39.09) | | 0.24 | | 0.46 | -30.22 (-58.65, -1.80) | | 0.04 | | 0.15 |
| 6’SL | 3.38 (-7.78, 14.55) | | 0.55 | | 0.65 | 24.42 (11.48, 37.35) | | 0.0003 | | 0.006 |
| DFLac | 4.32 (-7.05, 15.68) | | 0.45 | | 0.57 | -10.51 (-23.67, 2.65) | | 0.12 | | 0.20 |
| DFLNH | 2.14 (-2.26, 6.55) | | 0.34 | | 0.49 | 3.33 (-1.78, 8.43) | | 0.20 | | 0.27 |
| DFLNT | -23.71 (-54.04, 6.61) | | 0.12 | | 0.33 | -22.09 (-57.21, 13.03) | | 0.21 | | 0.27 |
| DSLNH | 1.84 (-1.82, 5.50) | | 0.32 | | 0.49 | 4.17 (-0.07, 8.41) | | 0.05 | | 0.15 |
| DSLNT | -3.29 (-9.20, 2.61) | | 0.27 | | 0.47 | -6.12 (-12.96, 0.73) | | 0.08 | | 0.19 |
| FDSLNH | 2.70 (-0.70, 6.10) | | 0.12 | | 0.33 | 3.20 (-0.73, 7.14) | | 0.11 | | 0.20 |
| FLNH | 3.90 (0.04, 7.77) | | 0.048 | | 0.32 | 5.01 (0.53, 9.49) | | 0.03 | | 0.15 |
| LnNT | -11.30 (-23.51, 0.92) | | 0.07 | | 0.32 | 2.32 (-11.83, 16.46) | | 0.75 | | 0.75 |
| LNT | -21.81 (-46.73, 3.11) | | 0.09 | | 0.32 | 18.28 (-11.58, 47.14) | | 0.21 | | 0.27 |
| LNFP I | -7.90 (-47.42, 31.63) | | 0.69 | | 0.72 | -45.18 (-90.95, 0.59) | | 0.05 | | 0.15 |
| LNFP II | -13.89 (-28.50, 0.78) | | 0.06 | | 0.32 | 13.00 (-3.96, 29.96) | | 0.13 | | 0.21 |
| LNFP III | 0.46 (-0.64, 1.55) | | 0.41 | | 0.55 | -0.52 (-1.78, 0.74) | | 0.42 | | 0.49 |
| LNH | 2.31 (-0.01, 4.64) | | 0.05 | | 0.32 | 0.89 (-1.80, 3.58) | | 0.51 | | 0.57 |
| LSTb | -0.33 (-2.13, 1.48) | | 0.72 | | 0.72 | -2.15 (-4.24, -0.06) | | 0.04 | | 0.15 |
| LSTc | -4.22 (-9.97, 1.54) | | 0.15 | | 0.33 | 1.83 (-4.83, 8.49) | | 0.59 | | 0.62 |

**Supplemental Table 2.** Multivariable linear regression analysis was used to examine the associations between PC1 and PC2 with HMO summary measures and HMO concentrations. Models adjusted for technical covariates (i.e., proportion of rRNA, volume of skim milk) as well as days postpartum, human milk collection time, breast feedings per day, maternal body mass index, maternal healthy eating index, and mother age.

^+^Diversity was estimated using Simpson’s Diversity measure.

**Supplemental Table 5.** Summary of the number of EV-miRNAs associated with HMOs measures and concentrations via multivariable linear regression analysis.

| **HMO Summary Measures** | **P < 0.05 Count** | **P_BH_ < 0.10 Count** |
| --- | --- | --- |
| Diversity | 23 | - |
| Sum of HMOs (nmol/mL) | 51 | - |
| HMO-bound sialic acid (nmol/mL) | 6 | - |
| HMO-bound fucose (nmol/mL) | 27 | - |
| **HMO Concentrations** | **P < 0.05 Count** | **P_BH_ < 0.10 Count** |
| 2’FL (nmol/mL) | 49 | 5 |
| 3FL (nmol/mL) | 62 | 17 |
| 3’SL (nmol/mL) | 28 | 1 |
| 6’SL (nmol/mL) | 52 | 1 |
| DFLac (nmol/mL) | 36 | 0 |
| DFLNH (nmol/mL) | 13 | 0 |
| DFLNT (nmol/mL) | 36 | 0 |
| DSLNH (nmol/mL) | 22 | 0 |
| DSLNT (nmol/mL) | 32 | 0 |
| FDSLNH (nmol/mL) | 26 | 0 |
| FLNH (nmol/mL) | 45 | 1 |
| LnNT (nmol/mL) | 16 | 0 |
| LNT (nmol/mL) | 28 | 1 |
| LNFP I (nmol/mL) | 28 | 1 |
| LNFP II (nmol/mL) | 20 | 0 |
| LNFP III (nmol/mL) | 7 | 0 |
| LNH (nmol/mL) | 57 | 4 |
| LSTb (nmol/mL) | 19 | 0 |
| LSTc (nmol/mL) | 12 | 0 |

**Supplemental Table 5.** Multivariable linear regression analysis was used to examine the associations between individual EV-miRNAs with HMO summary measures and HMO concentrations. Models adjusted for adjusted for technical covariates (i.e., proportion of rRNA, volume of skim milk) as well as days postpartum, human milk collection time, breast feedings per day, maternal body mass index, maternal healthy eating index, and mother age. “P < 0.05 Count” indicates the number of EV-miRNAs that were associated with each HMO measure based on P < 0.05. “P_BH_ Count” indicates the number of EV-miRNAs that were associated with each HMO measure based on P_BH_ < 0.10.

**Supplemental Table 6.** Top 5 most statistically significant putative pathways for EV-miRNAs significantly associated with HMO characteristics.

| **HMO Summary Measures** | **Pathway** | **FDR** |
| --- | --- | --- |
| Diversity | Pathways in cancer | 7.8x10^-11^ |
|  | Axon guidance | 9.6x10^-11^ |
|  | Ubiquitin mediated proteolysis | 1.5x10^-8^ |
|  | Focal adhesion | 6.4x10^-8^ |
|  | Signaling pathways regulating pluripotency of stem cells | 1.4x10^-7^ |
| Sum of HMOs (nmol/mL) | Axon guidance | 1.5x10^-12^ |
|  | Pathways in cancer | 1.5x10^-12^ |
|  | Autophagy – animal | 6.3x10^-9^ |
|  | Hippo signaling pathway | 9.0x10^-9^ |
|  | Ubiquitin mediated proteolysis | 1.7x10^-8^ |
| HMO-bound sialic acid (nmol/mL) | Pathways in cancer | 4.2x10^-6^ |
|  | Axon guidance | 5.8x10^-5^ |
|  | Hippo signaling pathway | 5.8x10^-5^ |
|  | Focal adhesion | 6.5x10^-5^ |
|  | Glutamatergic synapse | 6.5x10^-5^ |
| HMO-bound fucose (nmol/mL) | Axon guidance | 3.0x10^-12^ |
|  | Pathways in cancer | 3.0x10^-12^ |
|  | Hippo signaling pathway | 2.0x10^-7^ |
|  | Pl3k-Akt signaling pathway | 2.1x10^-7^ |
|  | Proteoglycans in cancer | 2.1x10^-7^ |
| **HMO Concentrations** |  |  |
| 2’FL (nmol/mL) | Axon guidance | 3.1x10^-9^ |
|  | Regulation of actin cytoskeleton | 3.1x10^-9^ |
|  | Pl3K-Akt signaling pathway | 4.2x10^-8^ |
|  | Pathways in cancer | 6.9x10^-8^ |
|  | FoxO signaling pathway | 8.6x10^-8^ |
| 3’FL (nmol/mL) | Pathways in cancer | 1.0x10^-9^ |
|  | Axon guidance | 1.2x10^-8^ |
|  | Ras signaling pathway | 1.2x10^-8^ |
|  | Rap1 signaling pathway | 2.2x10^-7^ |
|  | Glutamatergic synapse | 2.3x10^-7^ |
| 3’SL (nmol/mL) | Glutamatergic synapse | 2.4x10^-4^ |
|  | Circadian entrainment | 1.1x10^-2^ |
|  | Axon guidance | 3.3x10^-2^ |
|  | Cushing syndrome | 3.3x10^-2^ |
|  | Proteoglycans in cancer | 4.0x10^-2^ |
| 6’SL (nmol/mL) | FoxO signaling pathway | 1.4x10^-4^ |
|  | Regulation of actin cytoskeleton | 3.8x10^-4^ |
|  | Proteoglycans in cancer | 1.4x10^-3^ |
|  | cAMP signaling pathway | 1.4x10^-3^ |
|  | Circadian entrainment | 1.4x10^-3^ |
| FLNH (nmol/mL) | Signaling pathways regulating pluripotency of stem cells | 4.9x10^-2^ |
|  | MAPK signaling pathway | 4.9x10^-2^ |
| LNFP I (nmol/mL) | -- | -- |
| LNH (nmol/mL) | Proteoglycans in cancer | 3.4x10^-9^ |
|  | MAPK signaling pathway | 6.2x10^-7^ |
|  | Pl3K-Akt signaling pathway | 2.7x10^-6^ |
|  | Hepatocellular carcinoma | 1.5x10^-5^ |
|  | ErbB signaling pathway | 2.2x10^-5^ |

**Supplemental Table 6.** Putative pathways estimated using miRPath v4.0, KEGG pathway annotation, and microT-CDS and a MicroT threshold = 0.7.
