# Supplementary material for: Associations between human milk EV-miRNAs and oligosaccharide concentrations in human milk
Source: Front Immunol. 2024 Nov 20;15:1463463. doi: 10.3389/fimmu.2024.1463463 (PMC11614774; doi:10.3389/fimmu.2024.1463463)
Supplement: Supplementary file 6 [file Table5.docx]

**Supplemental Table 5.** Summary of the number of EV-miRNAs associated with HMOs measures and concentrations via multivariable linear regression analysis.

| **HMO Summary Measures** | **P < 0.05 Count** | **P_BH_ < 0.10 Count** |
| --- | --- | --- |
| Diversity | 23 | - |
| Sum of HMOs (nmol/mL) | 51 | - |
| HMO-bound sialic acid (nmol/mL) | 6 | - |
| HMO-bound fucose (nmol/mL) | 27 | - |
| **HMO Concentrations** | **P < 0.05 Count** | **P_BH_ < 0.10 Count** |
| 2’FL (nmol/mL) | 49 | 5 |
| 3FL (nmol/mL) | 62 | 17 |
| 3’SL (nmol/mL) | 28 | 1 |
| 6’SL (nmol/mL) | 52 | 1 |
| DFLac (nmol/mL) | 36 | 0 |
| DFLNH (nmol/mL) | 13 | 0 |
| DFLNT (nmol/mL) | 36 | 0 |
| DSLNH (nmol/mL) | 22 | 0 |
| DSLNT (nmol/mL) | 32 | 0 |
| FDSLNH (nmol/mL) | 26 | 0 |
| FLNH (nmol/mL) | 45 | 1 |
| LnNT (nmol/mL) | 16 | 0 |
| LNT (nmol/mL) | 28 | 1 |
| LNFP I (nmol/mL) | 28 | 1 |
| LNFP II (nmol/mL) | 20 | 0 |
| LNFP III (nmol/mL) | 7 | 0 |
| LNH (nmol/mL) | 57 | 4 |
| LSTb (nmol/mL) | 19 | 0 |
| LSTc (nmol/mL) | 12 | 0 |

**Supplemental Table 5.** Multivariable linear regression analysis was used to examine the associations between individual EV-miRNAs with HMO summary measures and HMO concentrations. Models adjusted for adjusted for technical covariates (i.e., proportion of rRNA, volume of skim milk) as well as days postpartum, human milk collection time, breast feedings per day, maternal body mass index, maternal healthy eating index, and mother age. “P < 0.05 Count” indicates the number of EV-miRNAs that were associated with each HMO measure based on P < 0.05. “P_BH_ Count” indicates the number of EV-miRNAs that were associated with each HMO measure based on P_BH_ < 0.10.
