# Supplementary material for: Associations between human milk EV-miRNAs and oligosaccharide concentrations in human milk
Source: Front Immunol. 2024 Nov 20;15:1463463. doi: 10.3389/fimmu.2024.1463463 (PMC11614774; doi:10.3389/fimmu.2024.1463463)
Supplement: Supplementary file 7 [file Table6.docx]

**Supplemental Table 6.** Top 5 most statistically significant putative pathways for EV-miRNAs significantly associated with HMO characteristics.

| **HMO Summary Measures** | **Pathway** | **FDR** |
| --- | --- | --- |
| Diversity | Pathways in cancer | 7.8x10^-11^ |
|  | Axon guidance | 9.6x10^-11^ |
|  | Ubiquitin mediated proteolysis | 1.5x10^-8^ |
|  | Focal adhesion | 6.4x10^-8^ |
|  | Signaling pathways regulating pluripotency of stem cells | 1.4x10^-7^ |
| Sum of HMOs (nmol/mL) | Axon guidance | 1.5x10^-12^ |
|  | Pathways in cancer | 1.5x10^-12^ |
|  | Autophagy – animal | 6.3x10^-9^ |
|  | Hippo signaling pathway | 9.0x10^-9^ |
|  | Ubiquitin mediated proteolysis | 1.7x10^-8^ |
| HMO-bound sialic acid (nmol/mL) | Pathways in cancer | 4.2x10^-6^ |
|  | Axon guidance | 5.8x10^-5^ |
|  | Hippo signaling pathway | 5.8x10^-5^ |
|  | Focal adhesion | 6.5x10^-5^ |
|  | Glutamatergic synapse | 6.5x10^-5^ |
| HMO-bound fucose (nmol/mL) | Axon guidance | 3.0x10^-12^ |
|  | Pathways in cancer | 3.0x10^-12^ |
|  | Hippo signaling pathway | 2.0x10^-7^ |
|  | Pl3k-Akt signaling pathway | 2.1x10^-7^ |
|  | Proteoglycans in cancer | 2.1x10^-7^ |
| **HMO Concentrations** |  |  |
| 2’FL (nmol/mL) | Axon guidance | 3.1x10^-9^ |
|  | Regulation of actin cytoskeleton | 3.1x10^-9^ |
|  | Pl3K-Akt signaling pathway | 4.2x10^-8^ |
|  | Pathways in cancer | 6.9x10^-8^ |
|  | FoxO signaling pathway | 8.6x10^-8^ |
| 3’FL (nmol/mL) | Pathways in cancer | 1.0x10^-9^ |
|  | Axon guidance | 1.2x10^-8^ |
|  | Ras signaling pathway | 1.2x10^-8^ |
|  | Rap1 signaling pathway | 2.2x10^-7^ |
|  | Glutamatergic synapse | 2.3x10^-7^ |
| 3’SL (nmol/mL) | Glutamatergic synapse | 2.4x10^-4^ |
|  | Circadian entrainment | 1.1x10^-2^ |
|  | Axon guidance | 3.3x10^-2^ |
|  | Cushing syndrome | 3.3x10^-2^ |
|  | Proteoglycans in cancer | 4.0x10^-2^ |
| 6’SL (nmol/mL) | FoxO signaling pathway | 1.4x10^-4^ |
|  | Regulation of actin cytoskeleton | 3.8x10^-4^ |
|  | Proteoglycans in cancer | 1.4x10^-3^ |
|  | cAMP signaling pathway | 1.4x10^-3^ |
|  | Circadian entrainment | 1.4x10^-3^ |
| FLNH (nmol/mL) | Signaling pathways regulating pluripotency of stem cells | 4.9x10^-2^ |
|  | MAPK signaling pathway | 4.9x10^-2^ |
| LNFP I (nmol/mL) | -- | -- |
| LNH (nmol/mL) | Proteoglycans in cancer | 3.4x10^-9^ |
|  | MAPK signaling pathway | 6.2x10^-7^ |
|  | Pl3K-Akt signaling pathway | 2.7x10^-6^ |
|  | Hepatocellular carcinoma | 1.5x10^-5^ |
|  | ErbB signaling pathway | 2.2x10^-5^ |

**Supplemental Table 6.** Putative pathways estimated using miRPath v4.0, KEGG pathway annotation, and microT-CDS and a MicroT threshold = 0.7.
